# Precision Medicine in Mite Allergic Rhinitis

**DOI:** 10.3389/falgy.2021.724727

**Published:** 2021-09-27

**Authors:** Ruperto González-Pérez, David El-Qutob, Antonio Letrán, Víctor Matheu

**Affiliations:** ^1^Allergy Department, Complejo Hospital Universitario de Canarias, San Cristóbal de La Laguna, Spain; ^2^Allergy Unit, University Hospital of La Plana, Villarreal, Spain; ^3^Allergy Unit, Hospital HLA Jerez Puerta del Sur, Jerez de la Frontera, Spain

**Keywords:** rhinitis-diagnosis, allergens-immunology, skin test allergens, challenge test, good clinical practice-GCP

## Abstract

It is well-known that a correct diagnosis is necessary for effective treatment. In the case of allergic rhinitis due to mites, imprecise diagnosis with effective but improvable methods means that in many cases an optimal result is not reached in patients. The diagnosis of allergic rhinitis due to mite sensitization have to require more homogeneously reproducible diagnostic tests that try to encompass many more of the protein antigens contained in them. With the few proteins that the problem has usually focused on, there is no they would cover many of the clinically relevant allergens in a large proportion of patients. In this mini-review we try to highlight the importance of having good allergenic sources and briefly gather information on various allergenic proteins included in mites that could be clinically relevant. All this to try to get closer to a more accurate diagnosis. We are also talking about two diagnostic tools that are clearly out of use and that should be promoted in the consultations to obtain an even greater and better outcome in patients.

## Introduction

Allergic rhinitis (AR) is the most common form of non-infectious rhinitis, with a profound financial burden and enormous impairment of quality of life and often associated with several comorbid atopic chronic conditions such as bronchial asthma and/or eczema (1–3). Despite the increase in allergic respiratory diseases has nearly doubled in the past two decades -with epidemiologic studies suggesting that 20–30% of adults and up to 40% of children are affected- AR is frequently ignored, misdiagnosed and inadequately managed (4, 5).

The clinical classical picture of persistent AR -characterized with 2 or more nasal symptoms such as nasal congestion, rhinorrhea, sneezing, and itching for more than 2 weeks- are the result caused by specific immunoglobulin E (sIgE) to inhalant allergens (6). In 1967, the physician Reindert Voorhorst and his coworkers at the University Hospital of Leiden, Netherlands, published a paper stating that the *Dermatophagoides* species mite was the main allergenic source in house dust set a milestone (7), not only solving the problem of the house dust allergen but also allowing the identification of this mite species in house dust samples from all over the world. From then on, these mites came to be known as house dust mites (HDM) remaining as a widespread and troublesome worldwide disease and there with an overwhelming amount of literature about the topic.

Distinctive attributes of dust mites have allowed them to colonize the indoor environment in most dwellings in the temperate regions of the world, thus producing a matchless assortment of allergens and adjuvants perfectly suited to induce both innate and adaptive immune reactions in sensitized individuals (8). Moreover, indoor allergens largely occupied by HDM are still considered to be the most important cause of sensitization in infants starting in young children even under 1 year of age (9). The most relevant sources of allergens in house dust are found in the fecal pellets of the mite species *Dermatophagoides pteronyssinus, Dermatophagoides farinae, Euroglyphus maynei*, and the storage mites *Blomia tropicalis, Lepidoglyphus destructor*, and *Tyrophagus putrescentiae* (10). The *Dermatophagoides* genus is the most investigated of all the HDMs, although species serological dominance varies, suggesting specialist geographical adaptation (11). This is the case for *Blomia tropicalis*, initially characterized as a storage mite and nowadays constituting an emerging key allergen, not exclusively restricted to the tropics (12).

## Diagnosis of Mite AR

Conventional AR diagnosis starts with a detailed anamnesis trying both to recognize the presence or absence of allergic symptoms and the eventual identification of the likely causative allergens. This may be generally simple in cases of isolated seasonal allergy but more complicated in polysensitized individuals afflicted with the persistent forms of the respiratory condition. In fact, a key feature of mite sensitization in certain areas of the world is the larger repertoire of specific mite allergens that the atopic individuals are sensitized to, possibly due to the presence of a more diverse group of mites being co-dominantly present in the environment (i.e., the concurrent presence of both *Blomia tropicalis* and *Dermatophagoides spp*. in the tropics) (13). Concerning the potential exposure to possible HDM allergens also a full social history is required, including housing conditions (floor level, dampness and mildew odors, dust reservoirs, carpeting, central air heating, or cockroach or rodent infestations), the existence of pets or contact with animals, and the characteristics of the work and/or school environment (14).

In subjects with symptoms suggestive of AR, further diagnostic testing is required to complete a full diagnosis and a more appropriate management. *In vivo* diagnosis of mite allergy in routine clinical practice relies on skin prick tests (SPT) with commercial extracts, which is nowadays considered the first-line interventional method to identify IgE mediated allergic diseases for patients with respiratory symptoms (15, 16). Skin prick test is reproducible, minimally invasive, relatively easy when properly performed, and allows the testing of a variable panel of reagents which generally depends on the prevalence of local aeroallergens (17).

Standardization of allergen vaccines/extracts are crucial to diagnose and treat mite allergy because their qualities are variable depending on production methods and manufactured lots with noticeable differences among countries (18). Standardized allergen extracts ideally should have a batch-to-batch consistency with the skin test results comparable when the same extracts from different manufacturers are used (19). In Europe, extracts are standardized using manufacturers' in-house references and labeled in manufacturer-specific units, while in the United States, allergen standardization is based on intradermal testing of allergic subjects and the potencies of lots are determined by inhibition of binding of IgE from pooled allergic sera to solid phase reference allergen extracts, or measurement of specific allergen contents in the allergen vaccines (20–22).

In this regard, previous studies have confirmed that certain natural *Dermatophagoides spp*. and *Blomia tropicalis* extracts lack important allergens showing a considerable variability in the allergen composition and content (23–25). Moreover, it has been shown that the concentration of major allergens correlates with the biological potency and IgE reactivity of allergen extracts (26). In our view, greater efforts should be made to implement a closer cooperation between allergen manufacturing companies and regulatory agencies to improve the overall quality and consistency of available mite extracts.

## Serodominant Allergens, Molecular Diagnosis

To date, the World Health Organization, and the International Union of Immunological Societies (WHO/IUIS)^1^ Allergen Nomenclature Sub-committee currently includes up to 39 *Dermatophagoides spp*. (Pyroglyphidae) allergens through specific IgE (sIgE) binding or skin test reactivity (WHO/IUIS allergen nomenclature sub-committee), with wide differences in the seroprevalence of the major Der p 1 -a 24 kDa cysteine protease- and Der p 2 -a ligand to TLR-4- allergens across regions (27). The 14 KDa peritrophin Der p 23 -present in the outer membrane of mite feces- has been described as the latest major HDM allergen inducing respiratory symptoms when the aforementioned, Der p 1, Der p 2, and Der p 10 are not recognized (28, 29).

Component-resolved diagnosis (CRD) for molecular diagnosis was firstly introduced in 1999, performing a multi-test allergen analysis enabling a comprehensive analysis of the patient's IgE-binding pattern to a wide number of individual allergens (30).

Although, a dominant role for sIgE sensitization to Der p 1, Der p2, and Der p23 has been well-described among individuals from different parts of the world with severe AR (31–33), further European and Asian studies reported that in temperate regions between 20 and 47% of 1,302 HDM allergic patients also showed sIgE to mid-tier and minor allergens -i.e., groups 4, 5, 7, 13, 15, and 21- explained by a different exposure to HDM probably determined by the local weather conditions (34, 35). As clinicians bear in mind the limitations of currently available mite diagnostic and therapeutic extracts, a wider knowledge -with CRD promoting a genuine molecular diagnosis- of the local population immunodominant dust mite allergens is warranted in order to describe a more precise sensitization profile of subjects afflicted with AR in different areas of the world.

Despite allergen immunotherapy (AIT) is effective, inexpensive and the only disease-modifying therapy for allergy, the development of AIT is also severely limited by the quality of natural allergen extracts and may only be achieved through molecular AIT strategies (36, 37). In fact, mite immunotherapy represents ~50% of the total volume of marketed vaccines, mainly of the genus *Dermatophagoides* (38).

The development of such molecular approach may play a direct role on specific therapy, as recently published by Rodríguez-Domínguez and coworkers, confirming that stratification of HDM allergic patients according to their molecular sensitization profiles -including molecular monitoring of AIT-induced IgG responses- may enhance the success of AIT (39). It has been recently pointed out that since CRD is a necessary step for future component resolved immunotherapy, the inclusion of components should be guided by the clinical impact of allergens, an aspect related to IgE-binding but not exclusively to IgE-binding frequency (40). The further development of new diagnostic and AIT tools, including cost-effectiveness molecular extracts and/or hybrid allergens, are needed to achieve a personalized management of dust mite AR patients, implementing more specific target therapies in the era of precision medicine.

## Nasal Provocation Test

Nasal allergen challenge (NAC) is also an important tool to diagnose allergic HDM rhinitis. The aim of NAC is to reproduce allergic nasal symptoms, such as sneezing, itching, nasal airway obstruction and nasal secretion, under standardized and controlled conditions (41), through exposure of nasal mucosa to an allergenic extract. There have been published several guidelines of NAC, explaining procedures and methods of evaluation (41–43). It is a test that can be used in routine clinical practice although must be conducted by properly trained personnel.

NAC is indicated in the diagnostic confirmation of allergic rhinitis, mainly in polysensitized patients, to evaluate the clinical relevance of allergens, when there are discrepancies between the clinical history and the skin and/or serological diagnostic tests. It is also important for assessing the nasal response with respect to the dose and response time after the application of allergens (44), and in research studies about physio-pathological mechanisms involved in the nasal response to allergens (2). Special mention should be made of local AR (LAR). LAR is a new phenotype of rhinitis characterized by a positive response to a NAC with local production of specific IgE, tryptase, and eosinophil cationic protein (ECP) in the absence of systemic IgE in serum, manifested by negative skin prick test and specific IgE (45).

The relationship between upper and lower airways, the so-called United Airway Disease, has been established through epidemiologic, pathophysiologic, and clinical studies, modifying the global pathogenic view of respiratory allergy (46). So, provocation by a nasal allergen induces inflammatory mediators in bronchial mucosa and sputum (7). There are studies in which NAC has been used to study the lower respiratory tract in patients with respiratory allergy to HDM (47). And some studies have shown the correlation -in around 70% of cases for HDM- between NPT and both SPT and sIgE in patients with a positive SPT (≥3 mm) or positive sIgE result (48). It has also been used (49). Therefore, NAC offers a safer alternative to bronchial provocation when evaluating the role of specific allergens in a patient's asthma.

NAC has no absolute contraindications, and it has been considered a low risk test (50). However, it should be avoided during pregnancy, and during the acute phase or exacerbation of a patient's allergic disease (rhinitis, food allergy, drug allergy, insect allergy, urticaria), or in patients with previous anaphylactic reactions to the allergen of interest (51). It is advisable to take precautions in patients with uncontrolled asthma, severe chronic obstructive pulmonary disease, or cardiopulmonary disease in which the use of adrenaline is contraindicated (41).

The standardization of allergen extracts is fundamental for the accuracy, safety, and reproducibility of any diagnostic procedure, included NAC. So, it is advisable to use only standardized HDM extracts. The dose and biological potency of the extract used in NAC may vary depending on the commercial laboratory, extract presentation, and delivery technique. HDM extracts for NAC are available in various forms such as solution or powder, and can be administered into the nasal cavity by several methods i.e., pump spray, paper disc, atomizer, pipettes, or dropper.

The response to HDM extract in NAC can be measured by subjective and objective methods (see Table 1). Initially, the HDM extract is instilled intranasally and the intensity of nasal symptoms such as itching, sneezing, rhinorrhea and nasal obstruction occurred are recorded. The objective evaluation of the nasal obstruction can be carried out by assessing the nasal flow with nasal peak of maximum inspiratory flow (NPIF), of the nasal resistances by rhinomanometry (RMN), and the geometry of the nostrils through the acoustic rhinometry. The most commonly method used today is active anterior RNM. It is a very sensitive and specific technique, but it cannot be performed in very obstructed patients and/or with important secretions, and it needs a good collaboration. PNIF has been used as an outcome parameter in research trials, both in natural conditions and in allergen exposure chamber (53). Due to its portability, simple application and good correlation to subjective symptoms, PNIF is a valuable tool for evaluation of NAC. Acoustic rhinometry (AR) is a quick, non-invasive, reproducible method, useful in the evaluation of nasal obstruction during NAC. In contrast to active anterior RMN, AR can be performed when one or both nostrils are totally blocked and thus it seems more suitable for the evaluation of bilateral NAC (54).

**Table 1:** Methods of measuring the nasal response to allergens (52).

Subjective methods	Anterior rhinoscopy	
	Evaluation of symptoms	Score
		Visual analog scale
Objective methods	Evaluation of nasal obstruction	NPIF
		Passive RMN
		Active RMN
		Acoustic rhinometry
	Evaluation of the nasal inflammatory response	Nasal cytology
		Determination of nasal specific IgE
		Determination of inflammatory mediators
		Nitric oxide measurement
	Evaluation of volume and weight of nasal secretions	
	Olfactometry	

We must take in account that this is a procedure does not exempt of false negative and false positive results. Possible causes of false-positive or false-negative test result are related to HDM extract used, and the examination conditions of the patient or site.

## Nasal Cytology

Nasal cytology (NC) represents an easy to perform, but unfortunately, underused in daily practice diagnostic-therapeutic procedure to define nasal inflammation profiles (stimulated naturally or by specific provocation test) (55). The technique is based on the quantification of cell population within the nasal mucosa when chronic rhinitis (allergic and non-allergic) or rhinosinusitis are suspected. It involves sampling, processing and microscope reading. After the sample is obtained (by nasal smear, swab, scraping, or irrigation) usually May-Grünwald-Giemsa staining is used to identify inflammatory nasal cells (in blue the nuclei of white blood cells and the granules of basophils while red blood cells and eosinophils granules are red). Then, the stained sample is read at optical microscopy with oil immersion (1,000× magnification) (56).

Although eosinophils and mast cells have been classically considered important markers of allergic inflammation (57), recently researchers have explored the pathogenic potential role of neutrophils in HDMs AR. Gelardi et al. (58) defined a group of 16 patients with persistent AR caused by monosensitization to HDMs were NC showed that the cells most detected in the nasal mucosa were neutrophils. During the period from October to April, a peak in the number of neutrophils and a presence of significant number of eosinophils, mast cells and lymphocytes cells were found, showing that in these months there is more intense inflammation. According to this, Ciprandi et al. (59) described “Minimal Persistent Inflammation” condition: In cases of a persistent and low intensity allergen exposition, like perennial AR by mites, persistent infiltration of neutrophils overall is presented and only minimally by eosinophils and mast cells. Instead, “the pollen forms” that are characterized by eosinophils and mast cells, which are mostly degranulated specially when samples are obtained during the pollen season (60).

It is also interesting how NC has become a useful instrument to clarify and distinguish non-allergic rhinitis (NAR) subtypes in HDMS AR (in cases of patients with no time correlation between symptoms and allergen season, the presence of atypical symptoms or not responsive to standard drug therapy) (61). An Italian real world multi-centre study investigated the role of NC in the workup of AR patients with HDM allergy. From forty-six patients with AR to HDMs, 45.7% (21 patients) also presented NAR (11 patients had eosinophil-mast cell non-allergic rhinitis-NARESMA, 6 neutrophilic non-allergic rhinitis-NARNA, 3 non-allergic with mast cell-NARMA and 1 eosinophilic non-allergic rhinitis-NARES) (62).

But NC is also important in the treatment of nasal inflammatory diseases. Recently, Chen et al. described 468 HDMs AR patients whose treatment was guided by NC. AR (Eos) and half of AR (Neu) were treated with mometasone furoate spray and loratadine. Another half of AR (Neu) were treated with clarithromycin. Minimal differences in clinical outcomes were discovered but authors recommended NC for subtyping AR patients optimizing an individual AR treatment (63).

## Final Summary

As conclusion, the diagnosis of mite allergy rhinitis goes beyond a couple of groups of allergenic proteins such as group 1 and group 2. The importance of evaluating more groups of proteins with clinical significance is already proven for group 23, as well as for groups 4, 5, 7, 13, 15, and 21 in the case of mites. Improvement of the tools for *in vivo* diagnosis using good extracts is urgent both for skin tests and for nasal provocation tests as well as *in vitro* procedures using good platforms of component-resolved diagnosis.

## Author Contributions

RG-P worked on allergens diagnosis. DE-Q worked on nasal provocation. AL worked on nasal cytology. VM conceived the idea and put all the manuscript in order. All authors contributed to the article and approved the submitted version.

## Conflict of Interest

The authors declare that the research was conducted in the absence of any commercial or financial relationships that could be construed as a potential conflict of interest.

## Publisher's Note

All claims expressed in this article are solely those of the authors and do not necessarily represent those of their affiliated organizations, or those of the publisher, the editors and the reviewers. Any product that may be evaluated in this article, or claim that may be made by its manufacturer, is not guaranteed or endorsed by the publisher.

## Footnotes

^1^WHO/IUIS allergen nomenclature sub-committee. *Allergen nomenclature* [Online]. Available online at: http://www.allergen.org (accessed May 16).
